# Acquired atresia of the external auditory canal and canaloplasty with Thiersch graft reconstruction: Outcomes and complications

**DOI:** 10.17305/bjbms.2022.7676

**Published:** 2023-01-06

**Authors:** Annalisa Pace, Valeria Rossetti, Giannicola Iannella, Irene Claudia Visconti, Alessandro Milani, Roberta Polimeni, Antonino Maniaci, Salvatore Cocuzza, Massimo Re, Giuseppe Magliulo

**Affiliations:** 1 Sense Organs Department, Sapienza University of Rome, Rome, Italy; 2 Surgical Sciences Department, Sapienza University of Rome, Rome, Italy; 3 Otorhinolaryngology Department, University of Catania, Catania, Italy; 4 Clinical Science Department, Polytechnic University of Marche, Marche, Italy

**Keywords:** Acquired atresia, external auditory canal, endoaural surgery, canaloplasty, skin graft

## Abstract

Acquired atresia of the external auditory canal (EAC) is a rare disease characterized by otorrhea and progressive hearing loss. Clinically, it is differentiated into two stages: the wet stage and the dry stage. The dry stage does not respond to pharmacological treatment and has to be treated surgically. One surgical option is canaloplasty of the EAC with Thiersch graft reconstruction. This study aimed to report the follow-up outcomes (otomicroscopic signs and pure tone audiometry [PTA]) in patients with acquired atresia treated with this technique. Eighteen adult patients surgically treated for acquired atresia of the EAC between 2010 and 2020 were enrolled. All underwent canaloplasty with Thiersch graft reconstruction by one senior surgeon. Otomicroscopy and PTA results were evaluated before and after surgery. Postsurgical follow-up was performed at 1–3–6–12 months and then annually. Presurgical otomicroscopic examination revealed stenosis that occluded more than 75% of the EAC in all patients, and preoperative PTA showed conductive hearing loss in 89% of patients. However, postsurgical otomicroscopic examination showed that 94% of patients had a normal EAC diameter after one year, and only one patient had anterior blunting and recurrent atresia. In addition, postsurgical PTA evidenced a normal range in 89% of patients after one year. In conclusion, acquired atresia of the EAC is a troublesome disease usually associated with hearing loss. Therefore, treatment is chosen to resolve its symptoms. The results demonstrate evidence that canaloplasty with Thiersch graft may be a suitable surgical method considering the lower incidence of recurrence and the excellent hearing outcomes.

## Introduction

Acquired atresia of the external auditory canal (EAC) is an uncommon disorder with different origins: chronic otitis, dermatologic diseases, benign/malignant neoplasms, traumatic and/or iatrogenic complications (previous radiotherapy or postsurgical treatment) [1]. Symptomatology is nonspecific and troublesome, especially with otorrhea and progressive hearing loss. Moreover, it could be linked with cholesteatoma development [2].

Clinically, it is differentiated into two stages: the wet stage (with otorrhea and fullness) and the dry stage. The wet stage can be treated with ear toilet, topical antibiotics, and steroid therapy or oral drugs. On the contrary, the dry stage is usually chronic and not responsive to pharmacological treatment [3]. Therefore, the surgical management has to be performed in the dry stage [4].

In 1966, Paparella first described canaloplasty covered with a thick skin graft as a possible surgical technique that is currently the most commonly used [3]. Moreover, caustication of eventual granulations may occur during follow-up or before surgical planning [5].

In the literature, only a few papers report outcomes after surgical treatment of acquired atresia of the EAC, methodically performing one single technique [6].

This study aimed to report the follow-up outcomes (otomicroscopic signs and pure tone audiometry [PTA]) in patients with acquired atresia treated with canaloplasty with Thiersch graft reconstruction.

## Materials and methods

Patients enrolled in this study were affected by acquired atresia of the EAC and were surgically treated by a senior surgeon (G.M) in our Department from 2010 to 2020. All of them underwent canaloplasty with Thiersch graft reconstruction. Exclusion criteria included: histological evidence of neoplastic lesion, the performance of only canaloplasty, and loss at follow-up.

Preoperative data collected were: the disease duration, symptoms, and otomicroscopic signs (grade of stenosis of the EAC). In addition, a preoperative computed tomography scan was always performed to evaluate eventual concomitant ear diseases.

Presurgical PTA was conducted, and auditory function was classified into: normal (0–20 dB hearing loss [HL]), mild hearing loss (20–40 dB HL), moderate hearing loss (40–70 dB HL), severe hearing loss (70–90 dB HL), and profound hearing loss (above 90 dB HL) [6].

Postsurgical follow-up was performed at 1–3–6–12 months and then annually. Patients that did not complete a follow-up of nearly one year were excluded. At each time point, otomicroscopic examination investigated the presence of eventual moist eczema, crusts, recurrent atresia, and anterior blunting. Moreover, PTA was repeated at each time point.

### Surgical technique

An endoaural approach was adopted, and an incision of 1–2 mm lateral to the fibrous plug was performed. The fibrous tissue was slipped from the anterior and posterior bony canal through blunt dissection. Posteriorly, the fibrous plug was lifted to the fibrous annulus. This maneuver allowed us to identify an avascular plane between the meatal fibrosis and the fibrous layer of the tympanic membrane. The dissection progressed to remove the entire lesion. Perforations of the tympanic membrane were more frequent in the anterior portion, as well as ossicular chain disruption, and they were fixed using myringoplasty and ossiculoplasty. The bony wall canal was surgically made cylindrical.

**Figure 1.**
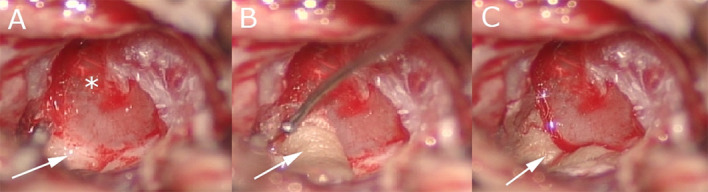
**Intraoperative view.** (A) Lack of skin bone covers after canaloplasty (white arrow) of the external auditory canal until the tympanic membrane (star); (B) positioning of Thiersch graft (white arrow) in the external auditory canal; (C) Thiersch graft (white arrow) correctly positioned.

Thiersch grafts enveloped the external auditory canal from the retro-auricular region (Figure 1) [7]. First, the dermo-epidermic graft sample was taken with a scalpel, and the dimension was variable based on the surface area to graft. Then, it was positioned on a stiff plate with the epithelium side facing down and cut into appropriately sized pieces removing eventual dermo-muscular residuals.

**Figure 2.**
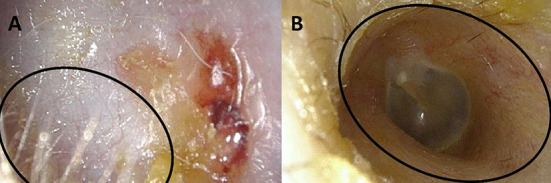
(A) Preoperative view: stenosis of the external auditory canal (EAC). The EAC is completely obliterated from the hair at the beginning (circle) toward the tympanic membrane (not visualized); (B) postoperative view: normal diameter of the EAC. The connection (circle) between Thiersch graft (with hair) and epidemic residue of the EAC (area without hair) is evident.

They were held in place by packing the ear canal with Gelfoam and with a thin sheet of Silastic. All patients were followed postoperatively. Standard protocol consisted of removing the packing after three weeks and then the patients underwent weekly control for four weeks. The patients were seen every three months during the first postoperative year (Figure 2).

### Ethical statement

Written consent was obtained from all patients. This study was completed following the Helsinki Declaration, approved by the Local Ethics Committee of “Sapienza” University (CE4324), and conducted according to good clinical practice.

### Statistical analysis

A descriptive statistical analysis was performed. Numerical variables were summarized as the mean, while categorical variables were represented as frequencies and proportions.

## Results

Thirty-two patients came to our Department with acquired atresia of the EAC. Five were excluded since Thiersch graft reconstruction was not performed, six did not complete follow-up, and three had a neoplastic histological diagnosis. Finally, 18 patients were considered for the study (7 males and 11 females; mean age: 56.9, range: 27–83).

The average disease duration was 3.8 years (range 1–7 years). The causes are reported in Table 1. An otomicroscopic examination revealed stenosis that occluded more than 75% of EAC in all patients. In three cases, the stenosis was associated with bleeding, and in eight cases, an external cholesteatoma was found.

**Table 1:** Otomicroscopic findings

**Ptn**	**Age**	**Sex**	**Side**	**Duration of disease (years)**	**Causes of acquired atresia of the EAC**	**Preoperative PTA**	**Postoperative PTA**			
							**1 month**	**3 months**	**6 months**	**1 year**
1	58	M	L	5	Iatrogenic	MoHL	MiHL		Normal	Normal
2	80	F	R	3	COM	M MoHL	M MoHL	M MiHL	M MiHL	M MiHL
3	45	M	R	6	COE	MoHL	MiHL	Normal	Normal	Normal
4	51	F	L	3	Idiopatic	MiHL	MiHL	Normal	Normal	Normal
5	58	M	L	4	Iatrogenic	MoHL	MiHL	Normal	Normal	Normal
6	43	F	R	1	COM	MoHL	Normal	Normal	Normal	Normal
7	83	F	L	4	COM	M SHL	M MoHL	M MoHL	M MiHL	M MiHL
8	59	M	L	5	Idiopatic	MoHL	MiHL	Normal	Normal	Normal
9	43	M	L	7	Idiopatic	MoHL	MiHL	Normal	Normal	Normal
10	77	M	R	4	COM	M SHL	MiHL	MiHL	Normal	Normal
11	61	F	L	3	Iatrogenic	SHL	Normal	Normal	Normal	Normal
12	50	F	L	4	COE	MoHL	MiHL	Normal	Normal	Normal
13	27	F	R	2	DD	SHL	MiHL	Normal	Normal	Normal
14	64	F	L	1	COE	MoHL	Normal	Normal	Normal	Normal
15	67	M	L	7	Iatrogenic	MoHL	MiHL	Normal	Normal	Normal
16	52	F	R	3	COE	MoHL	Normal	Normal	Normal	Normal
17	41	F	L	4	Iatrogenic	MoHL	MiHL	Normal	Normal	Normal
18	66	F	R	7	DD	SHL	Normal	Normal	Normal	Normal

Ptn: Patient; L: Left; R: Right; EAC: External auditory canal; PTA: Pure tone audiometry; COM: Chronic otitis media; COE: Chronic otitis externa; DD: Dermatologic disease; M: Mixed; N: Normal hearing; MiHL: Mild hearing loss; MoHL: Moderate hearing loss; SHL: Severe hearing loss.

Preoperative PTA showed conductive HL in 89% (16/18) of patients, while the others had mixed HL. The grade of HL is reported in Table 1. The otomicroscopic signs, evaluated during follow-up, are reported in Table 2.

**Table 2:** Otomicroscopic signs showed during follow-up. Each patient may present with simultaneously one or more signs. The number is referred to as the total sample (*n* ═ 18)

	**1 months**	**3 months**	**6 months**	**1 year**
Normal	5 (28%)	12(66%)	15 (83%)	17 (94%)
Moist eczema	15 (83%)	11 (61%)	4 (22%)	/
Crusts	8 (44%)	6 (33%)	1 (5%)	/
Recurrent atresia	/	/	1 (5%)	1 (5%)
Anterior blunting	/	1 (5%)	1 (5%)	1 (5%)

Histological analysis revealed one patient with dystrophic epidermolysis bullosa, whereas the others had chronic fibrosis.

Currently, 17/18 (94%) of patients at one year of follow-up, 1/18 reported recurrent atresia, and 1/18 reported an anterior blunting.

## Discussion

Acquired atresia of the EAC is an unusual disorder caused by diffuse, low-grade infection that could persist for months [8]. Risk factors are edema and infiltration of inflammatory cells [9], consequent to constant use of hearing aids that may provoke inflammatory status of the skin of the EAC and a cerumen stockpile [10].

The most frequently reported causes are chronic external otitis or dermatological diseases. This was evidenced in the series of Herdman and Wright [11] and Lin et al. [12], which described 100% and 85.7% of patients with these conditions, respectively. Our results align with these findings evidencing 94% of patients with an otitis history.

The treatment of this disease is correlated with the necessity to resolve the troublesome symptomatology, especially hearing loss [13].

Paparella and Kurkjian [5] first described the surgical approach to application in 1966. This was based on steps composed of removal of all connective tissue, preservation of fibrous layers of the tympanic membrane, widening of the bony EAC, and covering the bare bone and the tympanic membrane with skin grafts [14]. Although surgical treatment is the gold standard, it is frequently characterized by failures and recurrences.

Some publications examined outcomes of performing variety of surgical or follow-up techniques in a small sample of patients. Therefore, patients in this study were treated with one method performed by a single senior author to avoid bias. This author reported in 2009 the long-term results obtained with the same technique in 10 years of evaluation [7]. Magliulo reported 36% of recurrences in 5 years of follow-up. This is higher than the results obtained in the current study (5%). However, in this last series, there were no casually reported iatrogenic/radiotherapy or traumatic stenosis of the EAC [7].

Our surgical outcomes are encouraging since only two patients had a recurrence and anterior blunting after a one-year follow-up.

Our recurrence data are also lower than those of Schwarz et al., which presented a rate of 10.5%. They performed a different type of surgery, keeping the postoperative splinting of the anterior tympanomeatal angle with silicone sheets over three months. Moreover, the presence of silicone sheets may be a possible carrier for infection without a proper follow-up. On the other side, the PTA values were comparable [14].

PTA outcomes of our study were stable in all patients during follow-up. Moreover, 89% of patients reported normal hearing recovery after one year [15]. However, it is essential to note that the two patients with a partial benefit presented a mixed PTA curve before surgery.

This study had some limitations since it was designed as a first exploratory analysis to investigate the auditory outcomes of patients treated with canaloplasty and reconstruction in Thiersch graft for the stenosis of the EAC. However, based on these results, the authors intend to conduct larger sample studies to improve statistical power. However, it is to highlight that this disease remains rare, and it is hard to collect and follow patients. Moreover, there is a heterogeneity of causes of acquired stenosis of the EAC. Nevertheless, one author’s performance of the same technique is a point of strength that mantains the sample’s homogeneous. Further studies are underway to confirm the results.

## Conclusion

Acquired atresia is a rare disorder that can be treated mainly by surgery to re-establish patency of the EAC, since recurrence remains the most common complication. Unfortunately, not many studies on this topic can be found in the literature. This paper reports the otomicroscopic and auditory outcomes in a series of patients treated with a unique method performed by a single author. The rate of recurrence and PTA outcomes support this technique as a treatment to be proposed. Conversely, surgical success remains linked with subjective and etiological factors.

**Conflicts of interest:** Authors declare no conflicts of interest.

**Funding:** Authors received no specific funding for this work.

## References

[1] Hopsu E, Pitkaranta A (2007). Idiopathic, inflammatory, medial meatal, fibrotising otitis presenting with lichen planus.. J Laryngol Otol.

[2] Jotdar A, Dutta M, Kundu S, Mukhopadhyay S (2017). Advancing cholesteatoma secondary to acquired  atresia of  the  external  auditory canal: clinical perspectives.. J Clin Diagn Res.

[3] Pace A, Rossetti V, Visconti IC, Milani A, Iannella G, Maniaci G (2022 Sep 16). Thiersch graft follow-up with narrow band imaging for acquired atresia of the external auditory canal: canaloplasty with Thiersch graft versus vascularization evaluated with narrow band imaging.. Bosn J Basic Med Sci.

[4] Karkas A, Badidi G, Odinet P, Reynard P, Martin C (2019). Acquired medial external auditory canal stenosis, anterior tympanomeatal angle blunting, and lateralized tympanic membrane: nosology, diagnosis, and treatment.. Eur Ann Otorhinolaryngol Head Neck Dis.

[5] Paparella MM, Kurkjian JM (1996). Surgical treatment for chronic stenosing external otitis.. Laryngoscope.

[6] Brian C, KungThomas O, Willcox Jr (2007). Examination of hearing and balance.. Neurol Clin Neurosc.

[7] Magliulo G (2009). Acquired atresia of the external auditory canal: recurrence and long-term results.. Ann Otol Rhinol Laryngol.

[8] Roland PS (2001). Chronic external otitis.. Ear Nose Throat J.

[9] Hawke M, Jahn AF (1987). Chronic otitis externa. In: Diseases of the ear: clinical and pathologic aspects.. Philadelphia (PA): Lea and Febiger.

[10] Work WP (1950). XCI lesions of the external auditory canal.. Ann Otol Rhinol Laryngol.

[11] Herdman RCD, Wright JLW (1990). Surgical treatment of obliterative otitis externa.. Clin Otolaryngol Allied Sci.

[12] Lin VY-W, Chee GH, David EA, Chen JM (2005). Medial canal fibrosis: surgical technique, results, and a proposed grading system.. Otol Neurotol.

[13] Luong A, Roland PS (2005). Acquired external auditory canal stenosis: assessment and management.. Curr Opin Otolaryngol Head Neck Surg.

[14] Schwarz D, Luers JC, Huttenbrink KB (2018). Acquired stenosis of the external auditory canal—long-term results and patient satisfaction.. Acta Oto Laryngolica.

[15] Dhooge I, D’hoop M, Loose D, Acke F (2014). Acquired atresia of the external auditory canal: long-term clinical and audiometric results after surgery.. Otol Neurotol.

